# Cytogenetic profile of adult acute myeloid leukemia in Egypt: a single-center experience

**DOI:** 10.1186/s13039-022-00621-1

**Published:** 2022-10-05

**Authors:** Mohamed G. Elnaggar, Eman Mosad, Ahmed Makboul, Engy Adel Shafik

**Affiliations:** Clinical Pathology Department, South Egypt Cancer Institute, Assiut University, Assiut, 71515 Egypt

**Keywords:** AML, Karyotyping, Cytogenetics, Egypt

## Abstract

**Background:**

Acute myeloid leukemia (AML) is a diverse disease characterized by the expansion of blasts of myeloid lineage. Cytogenetic testing is the cornerstone for risk stratification of AML patients. Geographical and environmental factors may play a very important role in the development of leukemia and several differences in genetic profile may be seen among different ethnicities. In our study, we evaluated cytogenetic findings of adult AML patients in South Egypt.

**Methods:**

Cytogenetic testing (karyotyping and M-FISH) was performed for 120 adult patients with AML. Twenty metaphases were analyzed for each patient.

**Results:**

In our study, the median age of AML patients was 36.5 years, with an age range between 18 and 86 years. 56.7% of patients had normal karyotypes and 43.3% of patients had clonal cytogenetic abnormalities. t (15;17) was the most detected structural abnormality, and + 8 was the most detected numerical abnormality. Regarding cytogenetic risk stratification, 65% of patients were in the intermediate-risk category.

**Conclusion:**

The cytogenetic profile of AML patients in our locality showed some differences and some similarities with cytogenetic profiles in different Arab, Asian and Western countries. Further studies are needed using advanced techniques such as next-generation sequencing and optical genome mapping to elucidate more ethnic and geographic genetic heterogeneity among different countries.

## Background

Acute myeloid leukemia (AML) is a hematological neoplasm characterized by the uncontrolled increase of clonal, abnormally differentiated cells (i.e. blasts) in the bone marrow (BM), peripheral blood (PB), and possibly other organs [1]. AML is the most prevalent type of acute leukemia in adults, and its incidence rises with age. Understanding cytogenetic aberrations in AML is crucial for diagnostic and prognostic subtyping. It is also important for understanding the pathogenesis, potential clinical outcomes of patients, and treatment decisions [2]. Several studies showed that 50–60% of AML patients had cytogenetic aberrations [3, 4].

Geographical differences can result in cytogenetic heterogeneity in different hematological neoplasms. Furthermore, the occurrence of specific subtypes of AML in specific populations (such as acute promyelocytic leukemia in Latin populations or AML with t(8;21) in the Japanese population) supports the hypothesis that tumor-associated cytogenetic abnormalities in different hematological neoplasms may vary geographically and ethnically [5]. There is very little data regarding the cytogenetic profile of AML patients in Arab and African countries. Our study aims to report the cytogenetic profile of adult patients with de novo AML in South Egypt and to compare our results with the results from Western, Asian, and other Arab countries.

## Materials and methods

### Patients

This cross-sectional study included 120 adult patients with de novo AML, referred to South Egypt Cancer Institute, Assiut University, from 2019 to 2021.

### Ethical considerations

The Ethical Committee of South Egypt Cancer Institute, Assiut University approved our study (SECI-IRB IORG0006563—Registration number: 444). Before enrollment in our study, participants were asked to provide written informed consent. The study was registered on ClinicalTrials.gov (NCT number: NCT03719183). Our study conforms to provisions of the Declaration of Helsinki.

### Morphologic evaluation

The diagnosis of patients with AML was done by examination of BM aspirate smears. Patients were diagnosed with AML according to the 2016 World Health Organization (WHO) classification. Patients were morphologically subtyped into M0 through M7 according to the French-American-British (FAB) classification.

### Flow cytometric immunophenotyping

Flow cytometric immunophenotyping, using a panel of monoclonal antibodies, was performed for all patients in our study to confirm the diagnosis of AML.

### Cytogenetic analysis

Conventional cytogenetic analysis was done at diagnosis for all patients according to the standard techniques with G-banding [6]. First, BM cells were cultured without mitogens for 24 h in “MarrowMax™” BM medium. Afterward, colcemid was added to stop cell division at the metaphase. Following this, hypotonic treatment with potassium chloride was done for 25 min. Cells were then fixed with modified Carnoy’s fixative. Slides of metaphase chromosomes were prepared and were banded using the Giemsa trypsin banding (GTG) technique.

Metaphases were captured using Zeiss Axio Imager Z2 microscope **(Carl Zeiss GmbH, Jena, Germany)** and analyzed using Ikaros karyotyping software **(MetaSystems GmbH, Altlussheim, Germany).** Twenty metaphases or more were examined for each case. Karyotypes were defined according to the International System for Human Cytogenomic Nomenclature (ISCN) 2016 criteria.

### Multicolor FISH (M-FISH)

Multicolor FISH (M-FISH) was performed using “24Xcyte multicolor FISH probe” **(MetaSystems GmbH, Altlussheim, Germany) **[7]**.** Slides of metaphase chromosomes were prepared. After that, the slides were chemically denatured at 75 °C for 3 min. The slides were then dehydrated in ascending grades of alcohol (70%, 85%, and 100%). Afterward, the probe was placed on each slide and a coverslip was applied and sealed using rubber glue. The slides were placed in Hybrite **(Manufactured by Leica Biosystems Richmond for Dako Colorado Inc., Fort Collins, USA)**. The Hybrite was set at 80 °C for 5 min for denaturation, then at 37 °C for 48 h for hybridization. The slides were removed after completion of hybridization and washed. DAPI was then placed, and a coverslip was applied and sealed with nail polish. Images were captured using Zeiss Axio Imager Z2 microscope **(Carl Zeiss GmbH, Jena, Germany)** and they were analyzed using Isis software **(MetaSystems GmbH, Altlussheim, Germany).** At least 20 metaphases were analyzed for each case.

### Statistical analysis

Data were coded using the Statistical Package for the Social Sciences version 26 (SPSS Inc, Chicago, IL, USA). Categorical data were displayed as numbers and percentages, while continuous data were presented as median and range.

## Results

### Patient characteristics, morphology, and immunophenotyping of AML patients

The median age of AML patients was 36.5 years. Regarding gender, 64 patients (53.3%) were males and 56 patients (46.7%) were females with a male-to-female ratio of 1.14. The most frequent clinical presentation was organomegaly (hepatomegaly, splenomegaly, or both) in 37 patients (30.8%). The median WBC count was 42.3 × 10^9^/L.

Regarding morphological classification of AML, the most frequent subtype in our locality was AML with monocytic differentiation (AML-M4/M5) in 51.7% of patients followed by AML-M2 in 23.3% of patients (Table 1).

**Table 1:** Patient characteristics, morphology and immunophenotyping of AML patients

Total no of patients: 120 patients		
*1. Age group:*		
Median age	36.5 years	
Age range	18 – 86 years	
*2. Gender:*		
Male	64	53.3%
Female	56	46.7%
Male: female ratio	1.14	
*3. Clinical presentation:*		
Organomegaly	37	30.8%
Anemic manifestations	32	26.7%
Bleeding tendency	24	20%
Bone pain	18	15%
Fever	5	4.2%
Hypertrophied gum	3	2.5%
Lymph node enlargement	1	0.8%
*4. Hematological data:*		
Median WBC	42.3 × 10^9^/L	
*5. FAB subtypes*		
AML-M0	2	1.66%
AML-M1	15	12.5%
AML-M2	28	23.3%
AML-M3	11	9.2%
AML-M4	30	25%
AML-M5	32	26.7%
AML-M7	2	1.66%

*FAB*: French, American British, *WBC*: White blood cell; *AML*: Acute myeloid leukemia

### Cytogenetic profile of AML patients

Cytogenetic analysis was performed for all patients in our study. Regarding the results of cytogenetic analysis, 68 patients (56.7%) had a normal karyotype and 52 patients (43.3%) had an abnormal karyotype. t(15;17)(q24;q21) was the most common structural abnormality and it was detected in 11 patients (9.2%). This was followed by t(8;21)(q22;q22), inv(16)/t(16;16)(p13;q22) and t(v;11q23); each abnormality was detected in 9 patients (7.5%). inv(3)/t(3;3)(q21;q26) was found in 2 patients (1.6%). There was only 1 patient having t(9;22)(q34;q11) and 1 patient showed complex karyotype. Regarding numerical abnormalities, trisomy 8 was the most common numerical abnormality in 4 patients (3.3%), followed by trisomy 11 in 2 patients (1.6%) and − 7/del(7q) in only 1 patient. Other cytogenetic abnormalities are listed in Table 3.

Regarding cytogenetic risk stratification, patients were risk-stratified according to European LeukemiaNet (ELN) risk stratification [8]. 29 patients (24.2%) were categorized as favorable risk, 78 patients (65%) were categorized as intermediate risk, and 13 patients (10.8%) were categorized as adverse risk (Table 2).

**Table 2:** Cytogenetic risk stratification of AML patients

Risk category	No	Percent (%)
**Favorable risk**	**29**	**24.2**
t(8;21)(q22;q22)	9	7.5
inv(16)/t(16;16)(p13;q22)	9	7.5
t(15;17)(q24;q21)	11	9.2
**Intermediate risk**	**78**	**65**
Normal karyotype	68	56.7
t(9;11)(p12;q23)	1	0.8
Trisomy 8 (+ 8)	4	3.3
Trisomy 11 (+ 11)	2	1.6
del(3q)	1	0.8
der(1)t(1;6)(p36;q22)	1	0.8
i(17)(q10)	1	0.8
**Adverse risk**	**13**	**10.8**
t(v;11q23)	8	6.8
inv(3)/t(3;3)(q21;q26)	2	1.6
t(9;22)(q34;q11)	1	0.8
− 7/del(7q)	1	0.8
Complex karyotype	1	0.8

The bold values are signifying heading of the risk categories

*t*: Translocation; *inv*: Inversion; *i*: Isochromosome; *del*: Deletion

## Discussion

Acute myeloid leukemia (AML) is a hematologic neoplasm that consists of blasts of the myeloid lineage. The presence of at least 20% of blasts in the PB or BM is diagnostic [9]. The median age of AML patients in our study was 36.5 years (the age range was from 18 to 86 years). Several studies, including our study, found that AML patients were diagnosed at a younger age [10–13]. On the other hand, the median age of AML at diagnosis in Western countries varied from 61 to 71 years (older than the median age in our study). These results have been reported in developed countries such as the USA, United Kingdom (UK), Spain, Canada, and Australia [5, 14–19]. The younger age of AML patients in our study compared to Western countries could be attributed to differences in demographic characteristics, ethnicity, environmental, and genetic factors, which could play an important role in the development of AML at a younger age. There was a male predominance with 53.3% of patients in our study being males and 46.7% of patients were females with a male-to-female ratio of 1.14. This male predominance was in agreement with previous studies [5, 10–19]**.** Males tend to have a higher incidence of all leukemia in males due to their great exposure to work-related and environmental risks, according to some studies [20, 21].

Among the FAB subtypes, AML with monocytic differentiation (M4 and M5) represented the most prevalent FAB subtype and accounted for 51.7%, followed by AML-M2 with 23.3%. Our results agree with the studies of Mertelsmann et al. and van der Reijden et al., who reported AML-M5 as the most predominant subtype in their studies [22, 23]. Also, Abuhelwa et al. in Palestine reported that AML-M4 was the most prevalent subtype in their study and AML-M7 was the least common subtype [10]. On the other hand, several studies reported that the most prevalent subtype was AML-M2 [13, 24–28]. Chang et al. and Khoubila et al. reported that the most prevalent subtype in their studies was AML-M1 [12, 29]. Different populations may have different genetic backgrounds, which could explain why these results do not match up with what we found [30].

In our study, we reported cytogenetic findings in 120 adult patients with AML. Cytogenetic abnormalities were detected in 43.3% of patients, similar to that previously reported in literature ranging from 40 to 60% [25, 31]. We reported a normal karyotype as the most frequent cytogenetic finding in 56.7% of patients. Studies in Arab, Western, and Asian countries agree with our findings [11, 13, 25–27, 31–33]. Although AML with monocytic differentiation (M4/M5) was the most reported FAB subtype, most patients with normal karyotype were represented in this subtype (Table 3). Cases of normal karyotype AML are classified as intermediate risk. These cases are heterogeneous regarding response to treatment and relapse rate. They are affected by other genetic alterations, such as NPM1 mutation, CEBPA mutation, and other gene mutations that were not covered in our study. But in clinical practice, these molecular genetic studies should be part of the diagnostic workup, along with cytogenetic testing, so that patients’ risk groups can be categorized properly, and treatment outcomes can be improved.

**Table 3:** Distribution of cytogenetic findings among FAB subtypes

Cytogenetic findings	FAB subtypes						
	AML-M0	AML-M1	AML-M2	AML-M3	AML-M4	AML-M5	AML-M7
Normal karyotype	2	13	15	–	16	21	1
t(8;21)(q22;q22)	–	–	9	–	–	–	–
inv(16)/t(16;16)(p13;q22)	–	–	–	–	7	2	–
t(15;17)(q24;q21)	–	–	–	11	–	–	–
t(v;11q23)	–	–	–	–	5	4	–
inv(3)/t(3;3)(q21;q26)	–	1	–	–	–	1	–
t(9;22)(q34;q11)	–	–	–	–	–	1	–
Complex karyotype	–	–	–	–	–	–	1
Trisomy 8 (+ 8)	–	–	3	–	–	1	–
Trisomy 11 (+ 11)	–	–	1	–	–	1	–
i(17)(q10)	–	–	–	–	1	–	–
-7/del(7q)	–	–	–	–	1	–	–
del(3q)	–	1	–	–	–	–	–
der(1)t(1;6)(p36;q22)	–	–	–	–	–	1	–

*t*: Translocation; *inv*: Inversion; *i*: Isochromosome; *del*: Deletion; *FAB*: French American British

Regarding prognostic groups of AML patients in our study, they were classified into favorable (24.2%), intermediate (65%), and adverse (10.8%). In a study on the Moroccan population by Oum kaltoum Ait Boujmia et al., 17% of patients were favorable risk, 65.4% were in the intermediate-risk group, and 17.6% were adverse risk [13]. In another study by Khoubila et al., patients were classified into the favorable group (19.5%), intermediate (68%), and adverse group (12.5%) [12]**.**

**Fig. 1:**
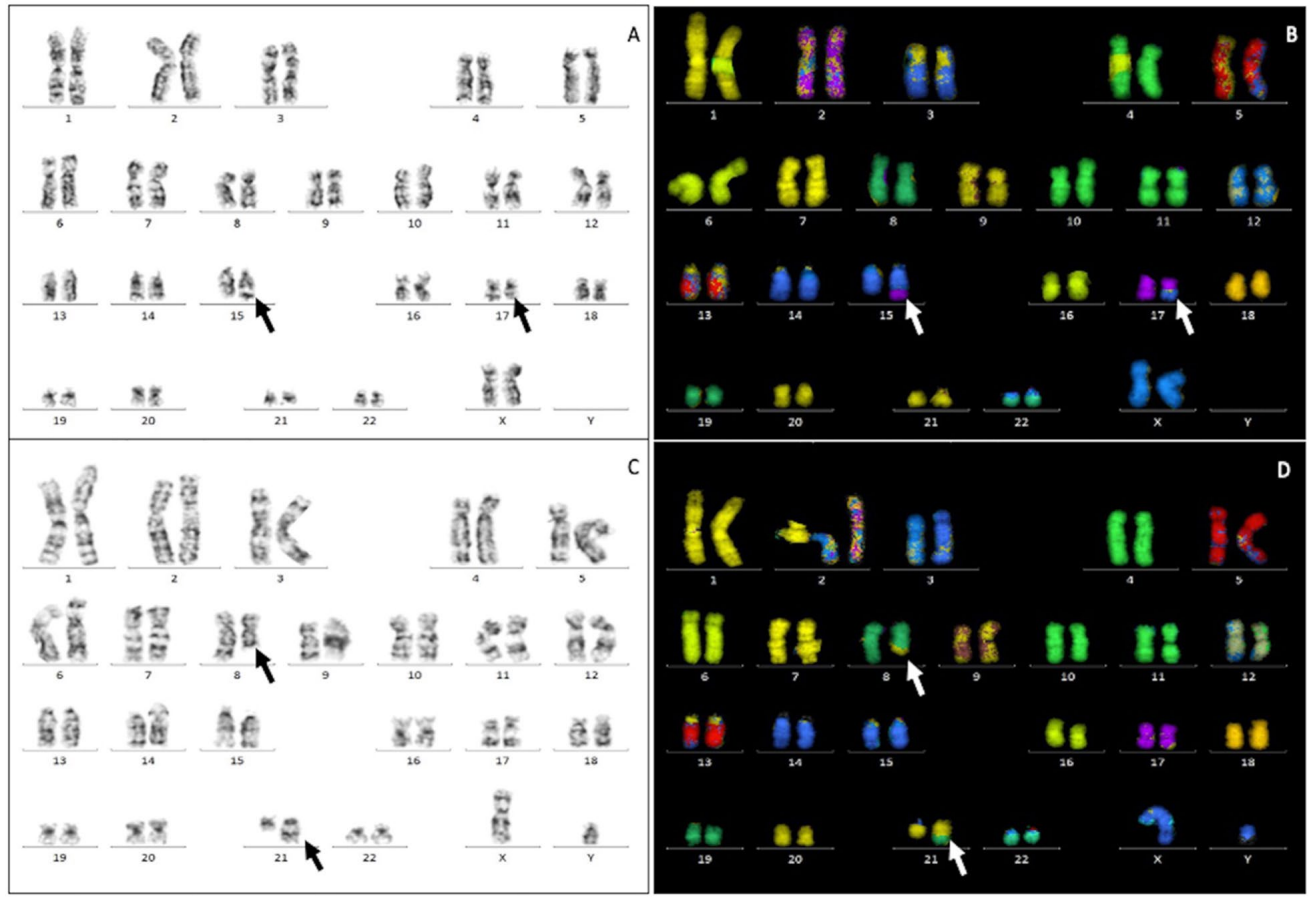
**A** t(15;17)(q24;q21) by G-banding **B** t(15;17)(q24;q21) by M-FISH **C** t(8;21)(q22;q22) by G-banding **D** t(8;21)(q22;q22) by M-FISH

In our study, t (15;17)(q24; q21) (Fig. 1A and B) was the most frequent structural abnormality in 9.2% of patients. This finding was also seen in Gmidene et al. study on the Tunisian population, which reported t(15;17) in 13.2% of patients [25]. In Western countries, t (15;17) was found to be the most common structural abnormality in 8% of the British population and 14.5% of the Spanish population, according to studies by Sanderson et al. and Sierra et al. respectively [5, 33]. In China, Cheng et al. reported t (15;17) as the most common abnormality (Table 4) [31].

**Table 4:** Comparison of cytogenetics of AML patients between our study and other studies worldwide

Cytogenetic finding	Frequency (%)											
	Our study 2021	Western population			Middle east population				Asian population			
		USA 2002 [34]	UK 2006 [33]	Spain 2006 [5]	Oman 2007 [27]	Tunisia 2012 [25]	KSA 2017 [11]	Morocco 2019 [12]	Morocco 2021 [13]	Japan 2008 [28]	China 2009 [31]	India 2020 [26]
Normal karyotype	56.7%	48%	45%	36%	38%	37.1%	36%	42%	44%	41.8%	42%	34.7%
*Structural abnormalities:*												
t(8;21)(q22;q22)	7.5%	8.7%	4%	2.7%	11%	12.2%	12%	12.5%	8.4%	17.7%	8%	20.8%
inv(16)/t(16;16)(p13;q22)	7.5%	7.9%	2%	2.7%	3%	3.8%	7%	3.3%	4.7%	4.1%	–	21.3%
t(v;11q23)	7.5%	4.5%	2%	3.3%	2%	3.5%	6%	1%	1%	5%	1%	3.4%
t(15;17)(q24;q21)	9.2%	–	8%	14.8%	10%	13.2%	6%	3.7%	3.9%	–	14%	8.6%
inv(3)/t(3;3)(q21;q26)	1.6%	1%	–	3.2%	–	–	2%	0.6%	0.2%	0.8%	–	3.4%
t(9;22)(q34;q11)	0.8%	–	1%	–	–	–	–	–	1.1%	1.1%	2%	–
Complex karyotype	0.8%	2.5%	15%	–	8%	10.8%	–	7.4%	12%	6.4%	6%	2.3%
*Numerical abnormalities:*												
Trisomy 8	3.3%	10.1%	6%	11.4%	11%	7%	15%	4.5%	5.3%	–	2%	–
Trisomy 11	1.66%	1.6%	1%	2.3%	–	–	–	–	3.3%	–	–	–
i(17)(q10)	0.8%	–	–	–	–	–	–	–	–	–	–	–
del(3q)	0.8%	–	–	–	–	–	–	–	–	–	–	–
Der(1)t(1;6)(p36;q22)	0.8%	–	–	–	–	–	–	–	–	–	–	–
-7/del(7q)	0.8%	7.8%	5%	8.6%	5%	3%	–	2.9%	2.7%	0.3%	1%	1.1%
del(5q)	–	3.5%	5%	9.1%	6%	2.2%	–	0.5%	–	0.3%	1%	2.3%

*AML*: Acute myeloid leukemia; *t*: Translocation; *inv*: Inversion; *i*: Isochromosome; *del*: Deletion

The frequency of t (8;21)(q22;q22) (Fig. 1C and D) in our study was 7.5%. Both Oum kaltoum Ait Boujmia et al. and Byrd et al. found that t(8;21) was reported in 8.4% and 8.7%, respectively [13, 34]. Other studies found a higher proportion of patients with t(8;21). In an Omani study, 11% of patients had t(8;21) [27]. Other research on Saudi and Moroccan populations found t(8;21) in 12% and 12.5% of patients, respectively [11, 12]. Another Japanese study found t(8;21) in 17% of patients [28]. An Indian study reported t(8;21) in 20.8% of patients (Table 4) [26].

Inv(16)/t(16;16)(p13;q22) was detected in 7.5% of patients in our study. This finding was consistent with the findings of Al Rajeh et al. in Saudi Arabia and Byrd et al. in the United States, which reported inv(16) in 7% and 7.9% of patients, respectively [11, 34]. On the other hand, the frequency of inv(16) was lower in studies in the UK [33], Spain [5], Tunisia [25], and Morocco [12, 13] while it was higher in an Indian study that reported 21.3% of patients with inv(16) (Table 4) [26].

Regarding numerical chromosomal abnormalities, trisomy 8 (+ 8) was the most frequent in our study. The clinical impact of additional copies of chromosome 8 on leukemic progression and responsiveness to treatment is debatable. Rather than being a primary cytogenetic abnormality, trisomy 8 is a disease-modulating secondary event. Therefore, gene expression analysis should be used to find out more about trisomy 8 in each AML subtype [35].

These differences between our study and other studies could be caused by genetic heterogeneity, ethnic differences, and environmental factors.

## Conclusion

In summary, effective treatment and supportive care are very important factors for the prognosis of AML patients, especially in countries with limited resources and limited available targeted therapies. Therefore, conventional cytogenetic analysis will remain the gold standard method for the detection of cytogenetic abnormalities and proper risk categorization for AML patients. Further studies on different populations and geographic regions can show the role of environmental and geographic factors in the development of AML. The cytogenetic profile in our locality showed some differences and some similarities with the cytogenetic profiles in different Arab, Asian and Western countries. Further studies are needed using advanced techniques such as next-generation sequencing (NGS) and optical genome mapping (OGM) to elucidate more ethnic and geographic genetic heterogeneity among different countries.

## Data Availability

The datasets used and/or analyzed during the current study are available from the corresponding author on reasonable request.

## Abbreviations

AML: Acute myeloid leukemia
BM: Bone marrow
DAPI: 4′,6-diamidino-2-phenylindol
del: Deletion
ELN: European LeukemiaNet
FAB: French–American–British
GTG: Giemsa trypsin banding
i: Isochromosome
inv: Inversion
M-FISH: Multicolor fluorescence in situ hybridization
NGS: Next generation sequencing
OGM: Optical genome mapping
PB: Peripheral blood
t: Translocation
UK: United Kingdom
USA: United States of America
WBC: White blood cell
